# The relationship between maternal COVID-19 with fetal and neonatal complications and intrauterine vertical transmission: A cohort study on pregnant women

**DOI:** 10.1371/journal.pone.0326450

**Published:** 2025-07-16

**Authors:** Zahra Shafizadeh, Zahra Akbarian-rad, Fatemeh Nasiri-Amiri, Mostafa Javanian, Hamid Reza Nouri, Mehdi Rajabnia, Zahra Geraili, Shanaz Barat, Jamileh Aqatabar Roudbari

**Affiliations:** 1 Student Research Committee, Babol University of Medical Sciences, Babol, Iran; 2 Amirkola Children’s Non-Communicable Diseases Research Center, Babol University of Medical Sciences, Babol, Iran; 3 Infertility and Health Reproductive Research Center, Health Research Institute, Babol University of Medical Sciences, Babol, Iran; 4 Infectious Diseases and Tropical Medicine Research Center, Health Research Institute, Babol University of Medical Sciences, Babol, Iran; 5 Cellular and Molecular Biology Research Center, Health Research Institute, University of Medical Sciences, Babol, Iran; 6 Infectious Diseases and Tropical Medicine Research Center, Health Research Institute, Babol University of Medical Sciences, Babol, Iran; 7 Social Determinants of Health Research Center, Health Research Institute, Babol University of Medical Sciences, Babol, Iran; 8 Infertility and Health Reproductive Research Center, Health Research Institute, Babol University of Medical Sciences, Babol, Iran; 9 Office of the vice-chancellor of the university, Department of Midwifery, Babol University of Medical Sciences, Babol, Iran; Shahid Beheshti University of Medical Sciences School of Medicine, IRANISLAMIC REPUBLIC OF

## Abstract

**Background:**

The clinical features, maternal and fetal complications, and the potential for vertical transmission of SARS-CoV-2 infection in pregnant women are still unknown. This cohort study aimed to determine the relation of coronavirus infection to the incidence of fetal and neonatal complications by comparing outcomes in pregnant women with COVID-19 to those in non-infected pregnant women.

**Method:**

This prospective cohort study was conducted on all pregnant women who gave birth between September 2020 to September 2021 at Rouhani Hospital, Iran. The case group included pregnant women infected with SARS-CoV-2, confirmed by clinical signs, symptoms and positive result of Real-time polymerase chain reaction (real-time PCR), who were followed up until 72 hours after birth. All fetal and neonatal complications, such as premature birth, oligohydramnios, fetal growth restriction, perinatal death and vertical transmission were recorded. Risk ratios for adverse pregnancy outcomes were analyzed by a modified Poisson regression model adjusted for relevant variables.

**Results:**

The final sample included 234 pregnant women (100 COVID-19 infected and 134 non-infected). The mean age was 29.42 ± 6.16 years (p = 0.12), and the mean BMI (body mass index) was 26.51 ± 4.16) Kg/m^2^ (p = 0.30). Cesarean delivery was more common among women with COVID −19 [adjusted risk ratio (aRR): 1.12, 95% confidence interval (CI): 1.09–1.25, p = 0.03]. A significant increase was observed in neonatal intensive care unit (NICU) admission in SARS-CoV-2 infected pregnant women compared to non-infected pregnant women [adjusted risk ratio (aRR):2.46, 95% confidence interval (CI): 1.06–5.42, P = 0.034]. Neonate born to women with COVID-19 had significantly higher rate of preterm birth (22% versus 11.2%) and low 5 minutes APGAR score (2% versus 0%) significantly (P < 0.05). Nearly all newborns tested negative for SARS-CoV-2 infection after birth (97.94%). Of 76 cord blood samples tested, 16 (21.05%) and 4 (5.26%) cases of infants born to mothers infected with COVID-19 had positive IgG and IgM antibodies, respectively.

**Conclusions:**

Neonates born to mothers with COVID-19 faced a significantly higher risk of cesarean delivery and NICU admission, but no significant differences in other pregnancy complications were observed between COVID-19 and non-infected groups, highlighting the complex effects of the virus on pregnancy outcomes.

## Introduction

Corona virus disease 2019 (COVID-19) is a type of highly contagious form of pneumonia, which World Health Organization (WHO) has declared as a global public health emergency. COVID-19 pneumonia was first reported in Wuhan, Hubei Province, China on December 31, 2019, followed by an outbreak in the entire Hubei Province and then other regions of the China [1,2]. On March 11, 2020, WHO declared COVID-19 as pandemic [3].

Corona virus is an enveloped virus with single-stranded RNA classified within the Coronaviridae family of the Nidovirales class [4].The coronavirus causes a wide range of respiratory and gastrointestinal symptoms in humans, from mild condition such as common cold to more severe illnesses such as bronchitis, pneumonia, acute respiratory syndrome (ARDS), coagulation disorders, multi-organ failure and even death [5–7]. Coronaviruses are classified into alpha and beta coronaviruses, which mainly infect mammals including bats, rodents, humans, and gamma and delta coronaviruses, which are mainly found in birds [8]. The potential of coronaviruses genome to rapidly mutate and undergo recombination leads to emergence of new coronaviruses with ability of crossing species barriers, resulting in zoonotic transmission [9,10]. Another coronavirus was emerged in Saudi Arabia in 2012, caused Middle East respiratory syndrome (MERS), which was transmitted from camels to humans [11,12]. SARS-CoV-2, the virus caused an outbreak worldwide, belongs to lineage B of the subgenus sarbecovirus [13]. The genome sequence analysis shows that SARS-CoV-2 the shares 87% −89% nucleotide identity with bat-related coronaviruses [14]. The main cause of the outbreak has been the transmission from individuals infected with the SARS-CoV-2 regardless of whether the individuals are symptomatic or asymptomatic through respiratory droplets or direct contact [13].

Several studies were conducted on patients with COVID-19 pneumonia in terms of epidemiological, clinical, laboratory and radiological characteristics, as well as therapeutic and clinical outcomes [2]. However, the clinical features, maternal and fetal complications, and the potential of vertical transmission of COVID-19 pneumonia in pregnant women remain largely unknown [15].

Pregnant women are susceptible to coronavirus infections due to the physiological changes of pregnancy, which can pose risks to maternal and fetal health [16]. If pregnant women develop viral pneumonia, there is a possibility of complications and progression to severe disease [17]. Symptomatic COVID-19 can mimic the signs of severe preeclampsia during pregnancy [18]. A systematic review and meta-analysis shows that women with SARS-CoV-2 infection during pregnancy had a significantly higher odds of developing preeclampsia than those without SARSCoV-2 infection during pregnancy [19]. However, not all studies are aligned on this issue. In some studies, COVID-19 infection has not been associated with an increased risk of preeclampsia [20]. Some studies have shown that the impact of the COVID-19 pandemic on pregnancy outcomes is similar to that observed in uninfected pregnant women [21].

Also regarding the transmission of the SARS-CoV-2 from mother to baby and subsequent neonatal complications after birth, finding have been inconsistent in this context. In one case, an infant born to a mother with COVID-19 had abnormal antibody levels and cytokine (IL-6, 28.26 pg/mL; IL-10, 153.60 pg/mL)results just 2 hours after birth, supporting the possibility of vertical transmission [22]. However, another study reported very diverse symptoms in pregnant women suffering from COVID-19 pneumonia, but found no evidence of virus transmission from mother to fetus and newborn [23].

An important unanswered question is whether SARS-CoV-2 can be transmitted from a pregnant woman to her fetus (a process called vertical transmission) and, if so, what the mechanism of transmission might be [24,25]. This question is very important given the history of vertical transmission in newly emerging viral infections such as Zika virus, Ebola virus and Marburg virus, which have posed significant risk to both maternal and fetus health. In addition, answering this question is necessary for formulating treatment principles for pregnant women with COVID-19 [26–28]. Since COVID-19 is a new disease and its complications on babies born to infected mothers is unknown, this study was prospectively designed to determine the relation of coronavirus infection to the incidence of fetal and neonatal complications by comparing outcomes in pregnant women with COVID-19 to those in non-infected pregnant women at educational and medical centers of Babol University of Medical Sciences.

## Methods

### Study design and participants

This study was designed as a cohort and was conducted at Rouhani Hospital, an academic hospital affiliated to Babol University of Medical Sciences. This study has been approved by the ethics committee of Babol University of Medical Sciences. The ethical code is IR.MUBABOL.REC.1399.115. Informed consent was obtained from all participants in this study. All pregnant women admitted to Rouhani Hospital between September 2020 and September 2021 were assessed in term of inclusion and exclusion criteria. Available sampling was used to collect the samples. The sample size was determined based on the number of variables studied and the correlation between the variables [29], resulting in 100 pregnant women in each group and a total of 200 samples.

First, all pregnant women hospitalized in the midwifery and labor department of the hospital were divided into two groups based on the presence or absence of clinical symptoms of COVID-19 infection. One group included of pregnant women who had clinical signs and symptoms of the COVID-19, while the other group included those without any clinical signs and symptoms. The clinical symptoms of COVID-19 comprised fever, dry cough, progressive cough, tachypnea, shortness of breath, diarrhea, anosmia, decreased oxygen saturation and fatigue. All pregnant women admitted to the midwifery and labor department were tested for SARS-CoV-2 using reverse transcriptase polymerase chain reaction (RT-PCR). Women with both clinical symptoms of COVID-19 and a positive RT-PCR result were classified as an infected group, while women with negative RT-PCR results and no clinical signs and symptoms of COVID-19 were considered as the control group. “Patients who presented with symptoms of COVID-19 but tested negative in the RT-PCR were excluded from our study; however, according to the hospital protocol, they were retested due to the persistence and severity of clinical symptoms of COVID-19 and were provided with appropriate treatment.”

To minimize the presence of individuals without clinical symptoms of COVID-19 in the control group, a strict exclusion method was used. Those who tested positive for SARS-CoV-2, but did not have clinical signs and symptoms of COVID-19 were excluded from the study. In addition, the other exclusion criteria for the control group included a history of suspected clinical symptoms of the COVID-19 in the past 14 days, recent contact with infected or suspected individuals within the past 2 weeks in their family, presence in crowded areas such as shopping malls or parties as well as travel within in the past 14 days. Women who had multiple pregnancies were excluded from the study.

The case group included of pregnant women who were infected with corona virus based on clinical signs and symptoms and positive RT-PCR result, and were monitored up to 72 hours post-delivery. The concurrent control group included pregnant women admitted to the same hospital during the time of the investigation, but revealed no signs and symptoms of infection and had negative RT-PCR result for SARS-CoV-2. All fetal complications such as premature birth, oligohydramnios, fetal growth restriction, perinatal death and intrauterine vertical transmission were recorded.

### Procedures

Patients were treated according to WHO protocols. All other managements and procedures for both groups were performed based on obstetric indications and our hospital’s protocols. Due to the lack of strong evidence to caesarean delivery for SARS-CoV-2 infected women, the hospital’s protocol dictated that the method of delivery be determined based on other gynecological and obstetric indications. Additionally, it should be mentioned that women spend labor and delivery and postpartum (LDR) in separate rooms.

During the pandemic, we decided to avoid using common tools, such as birth balls and labor and water birth methods. In addition, physicians, residents, and midwives who care of pregnant women used patient specific equipment and avoid attending to other patients simultaneously. Complete personal protective equipment is used for the care of babies born to infected mothers.

All neonate born to infected mothers were separated from their mothers immediately after birth. The COVID-19 RT-PCR was performed on nasopharyngeal sample within the first 24 hours after birth, if neonate revealed clinical symptoms such as temperature instability, respiratory distress, runny nose, cough, reluctance to breastfeed, drowsiness, gastrointestinal symptoms such as vomiting.

A brief overview of the procedure and its significance: The healthcare provider gathers necessary materials, including a sterile swab and collection tube, and ensures a clean environment. Positioning: The newborn is typically placed in a safe and comfortable position, often on a flat surface. Swabbing: The provider gently inserts the swab into one of the newborn’s nostrils, aiming towards the nasopharynx (the area at the back of the nose). The swab is rotated gently to collect secretions, then withdrawn carefully. Sample Handling: The swab is placed into a sterile container and labeled for testing. It is crucial to handle the sample according to biohazard protocols. Testing: The sample is sent to a laboratory for PCR to determine if the newborn is infected with COVID-19. If a newborn tests positive, healthcare provider can implement monitoring and treatment protocols to manage potential complications. Parents are usually informed about the procedure and may need to provide consent.

### Evaluation of specific IgG and IgM antibodies against SARS-CoV-2 in newborns

Blood collection immediately after birth is crucial for assessing the trans placental transfer of maternal antibodies to the newborn. In this study, 10 ml (milliliter) of blood was collected from the umbilical cord, a procedure performed after clamping and cutting the cord post-delivery. Umbilical cord blood sampling facilitates the evaluation of neonatal immune responses and determines whether maternal SARS-CoV-2 infection has conferred immunity to the newborn. The collected blood samples were promptly transported to the hospital laboratory to ensure sample integrity and accurate analysis. Specific immunoglobulin G (IgG) and immunoglobulin M (IgM) antibodies against SARS-CoV-2 were assessed using an enzyme-linked immunosorbent assay (ELISA). IgG antibodies indicate prior infection or exposure, reflecting potential protective immunity transferred from the mother, while IgM antibodies typically signify a recent infection and the activation of the acute immune response. The ELISA tests were performed according to the manufacturer’s protocol provided by Pishtazteb (Tehran, Iran) to ensure standardization and reliability of the results. Antibody concentrations were interpreted using established cut-off values. Positive: Values >1.1, indicating the presence of SARS-CoV-2 antibodies in the neonate. Negative: Values <0.9, indicating no detectable antibodies and the absence of an immune response to SARS-CoV-2. Inconclusive: Values between 0.9 and 1.1, necessitate further testing to clarify the findings. SARS-CoV-2 antibodies in newborns provide important insights into the effectiveness of maternal immunity and the potential for vertical transmission of the virus. Positive results for IgG or IgM antibodies underscores the need for careful monitoring of newborns from SARS-CoV-2-infected mothers, while negative or inconclusive results warrant additional evaluation. In this study, only positive IgG or IgM results were considered as evidence of SARS-CoV-2 transmission from mother to fetus.

### Data collection

The tools used in this study include a checklist based on study variables, the perinatal file of pregnant mothers, labor and delivery records and a newborn examination conducted by a neonatologist on the first day of birth. Cord blood samples were taken from infants who were born to mothers infected with Covid-19 to assess the presence of IgG and IgM antibodies. Demographic data and clinical characteristics, comorbidities, laboratory and radiological findings at admission, as well as pregnancy and neonatal outcomes, were extracted using a checklist. Medical records and collected data were reviewed and retrieved by a trained team of researchers.

### Statistical analysis

The descriptive data of the patients were reported using the central index of the mean and the dispersion index of the standard deviation or as numbers and percentages. To determine the relationship between prenatal and neonatal outcomes in two groups, chi-square test was used between two classified variables. If the conditions of chi-square test were not met, Fisher’s test was used. Student’s t-test was applied for continuous variables. Risk ratios (RRs) for adverse pregnancy complications and neonatal outcomes, with a 95% confidence interval, were estimated using a modified Poisson regression model. In addition, a linear mixed effects model was used to analyze changes, adjusting for potential confounding factors such as mother’s age, mother’s body mass index, gestational age, number of deliveries, previous pregnancy complications, coexisting of other diseases, and mode of delivery. Statistical analysis was performed using Stata 16.0 (Stata Corp, College Station, TX, USA). All statistical tests were two-tailed, with a significant level set at the P < 0.05.

## Results

During the study period, 335 pregnant women were visited to Rouhani hospital with respiratory complaints and were evaluated by an infectious disease specialist. Among them, 180 individuals were suspected to SARS-CoV-2 infection and subjected to RT-PCR diagnostic testing. Of these, 129 were hospitalized, and 29 either did not meet the inclusion criteria or declined participation in this study. Overall, 100 pregnant women with confirmed COVID-19 were included in the study. In addition, 134 uninfected pregnant women who were hospitalized during the same time were included in this study as a control group based on strict eligibility criteria. The demographic and clinical characteristics of pregnant women in SARS-CoV-2 infected and uninfected groups are shown in Table 1.

**Table 1:** Demographic- fertility characteristics of COVID-19 infected and control groups (n = 234).

Variables	Infected pregnant women(n=100)	Non -infected pregnant women(n=134)	Total (n=234)	P-valuec
Age (year)^a^	30.14 (5.5)	28.89 (6.60)	29.42 (6.16)	0.120
BMI* (kg/m^2^ )^a^	26.20 (3.50)	26.70 (4.60)	26.51(4.16)	0.300
Weight at Birth (gr) ^a^	3088.86 (629.90)	3055.13 (556.30)	3069 (587.29)	0.660
**Grade education** ^**b**^	** **			0.315
High school	21 (21.00)	39 (29.1)	60 (25.6)	
Diploma	54 (54.00)	61 (45.5)	115 (49.1)	
University	25 (25.00)	34(25.4)	59(25.2)	
**Job** ^**b**^	** **			0.250
Housewife	93(93.00)	120 (89.6)	213 (91.00)	
Employee	7 (7.00)	14 (10.4)	21 (9.00)	
**Gravidity** ^**b**^				0.400
1	32 (32.00)	46 (34.30)	78 (33.30)	
≥2	68 (68.88)	88 (65.70)	156 (76.70)	
**Gender of the baby** ^**b**^				0.121
Boy	56 (56.00)	65 (48.50)	121 (52.20)	
Girl	42 (42.00)	69 (51.50)	111 (47.8)	
**Abortion history** ^**b**^				0.810
None	74 (74.00)	94 (70.10)	168 (71.80)	
1	20 (20.00)	31 (23.10)	51 (21.80)	
≥2	6 (6.00)	9 (6.70)	15 (6.40)	
**Comorbidity** ^**b**^	30 (30.00)	23 (17.2)	53 (22.6)	**0.016**

BMI, body mass index. ^a.^Values given as mean (SD), ^b.^Values given as number (%), ^c.^Continuous variables compared with independent t-test, categorical variable compared with chi-square test or Fisher’s exact test.

### Demographic and fertility characteristics in two groups

The age range of the participants in our study was 17 to 45 years and their mean age was 29.42 ± 6.16 years. Two groups of infected and non-coronavirus infected mothers did not have any significant difference in terms of age. The mean body mass index of women participating in this study was 29.42 ± 6.1 years old, and there was no significant difference between the two groups. All of participating women were in the third trimester of pregnancy and the average gestational age at the time of visiting the hospital was 35.71 ± 5.75 weeks. The minimum gestational age was 28 weeks and the maximum was 41 weeks. In terms of gestational age, the two groups had a statistically significant difference, so that in non-infected women, the mean gestational age in women referred to the prenatal clinic was 37.70 ± 2.00 weeks, but in women infected with Corona, it was 33.10 ± 7.70 weeks. Women infected with Covid-19 had a lower gestational age (P = 0.001).

There was no difference between the groups in terms of education level, occupation, number of pregnancies, gender of the baby and birth weight of the baby, but pre-existing medical problems including hypertension, diabetes**,** thyroid disorders, cardiovascular diseases, autoimmune disorders and others, the two groups had statistically significant differences. Corona infected women had more comorbidity associated with pregnancy (P = 0.016). (Table 1). To avoid its confounding effect, unadjusted and adjusted risk estimates on maternal and neonatal outcomes are presented in Table 5.

### Clinical and laboratory findings of infection in pregnant women infected with Covid-19

Overall, 97 cases of SARS-CoV-2 infected women continued their pregnancy until the end of the study, and two women presented with still birth, and one woman suffered intrauterine fetal death at the labor with 36th week of pregnancy. As shown in Table 2, fever (69%) was the most common symptom observed. A total of 22 (22%) patients had decreased oxygen saturation. The most frequent abnormalities in laboratory tests included an increase in CRP (62%). leukocytosis (12%) and lymphopenia (6%) (Table 3). Moreover, during our study, six pregnant women with covid-19 (6%) were admitted to the intensive care unit (ICU) and all of them had hypoxia and they required intubation and invasive ventilation. In late August 2021, a very ill 26-year-old pregnant woman with no underlying disease who had been infected by COVID-19 virus was referred to our hospital at 36 gestational weeks because of fever and chilling, severe shortness of breathing and dry cough that had started seven days earlier. She developed lymphopenia, thrombocytopenia, high C-reactive protein (CRP), increased liver enzymes (Alanine Aminotransferase (ALT) and Aspartate Aminotransferase (AST) and low O2 saturation (65%) and unfortunately died due to respiratory distress syndrome, acute damage to the liver system and acute kidney damage. But the female fetus was removed immediately by cesarean section and a girl was born with an Apgar score of 6–7 in the first minute and 8–9 in the fifth minute. She came and was immediately admitted to the neonatal intensive care unit and was discharged after 4 days in a good condition. In the women of the control group, there was no hospitalization in the intensive care unit. The number of patients hospitalized in ICU was significantly higher than the control group (P < 0.001). The reason for the mothers’ admission to the ICU was due to COVID-19, in all cases (Table 4).

**Table 2:** Sign and symptoms and clinical feature of COVID-19 infected pregnant women (n = 100).

Variable	Number (%)
Fever	69 (69)
Chill	59 (59)
Nausea	15 (15)
Vomiting	6 (6)
Diarrhea	6 (6)
Headache	34 (34)
Myalgia	63(63)
Through	30 (30)
Abdominal pain	10 (10)
Dry cough	46 (46)
Dyspnea	48 (48)
Short breath	37 (37)
Fatigue	52 (52)
Sleepy	19 (19)
Sneezing	3 (3)
Runny nose	11 (11)
Smell disorder	2 (2)
Dizziness	2 (2)
Body pain	2 (2)
Itching	1 (1)

**Table 3:** Comparison of laboratory symptoms in two groups COVID-19 infected and control groups (n = 234).

laboratory symptoms	infected pregnant women(n = 100)	Non--infected pregnant women(n = 134)	P-value^c^
White blood cell count (×10^9^/l)			<0.001
4000-16000	82(82)	133 (99.25)	
<4000	6 (6)	1 (0.75)	
>16000	12 (12)	0 (0)	
Lymphocyte count (×10^9^/l)			<0.001
200-1000	66 (66)	134 (100)	
<200	33 (33)	0 (0)	
>1000	1 (1)	0 (0)	
Blood platelet count (×10^9^/l)			<0.004
150-450	90 (90)	132 (98.50)	
<150	10 (10)	2 (1.50)	
C-reactive protein (mg/l)			<0.001
0.5-10	38 (38)	134 (100)	
10-120	62 (62)	0 (0)	
ALT^a^ (U/L)			<0.001
<31	94 (94)	134 (100)	
>31	6 (6)	0 (0)	
AST^b^ (U/L)			<0.001
<31	94 (94)	134 (100)	
>31	6 (6)	0 (0)	
Hb^c^ (gr/dl)			0.1
Nov-14	97 (97)	130 (97.01)	
<11	2 (2)	2 (1.49)	
>14	1 (1)	2 (1.49)	

^a^ALT, Alanine Aminotransferase; ^b^AST, Aspartate Aminotransferase;^c^ Hb, hemoglobin

Continuous variables compared with independent *t*-test, categorical variables compared with chi-square test or Fisher’s exact test.

**Table 4:** Comparison of pregnancy and neonatal outcome in two groups COVID-19 infected and control groups (n  =  234).

Pregnancy and neonatal outcome	*infected pregnant women*(n = 100)	Non-*-infected pregnant women*(n = 134)	P- value^c^
Still birth	2 (2.00)	0 (0)	0.182
IUFD at labor	1 (1.00)	0 (0)	0.427
Pre-eclampsia	7 (7.00)	8 (6.00)	0.476
PIH	2 (2.00)	1 (0.70)	0.391
IUGR	5 (5.00)	7 (5.20)	0.593
Gestational diabetes	17 (17.00)	16(11.90)	0.181
Maternal death	1 (1.00)	0 (0)	0.427
PPH	1 (1.00)	0 (0)	0.427
Maternal ICU admission	6 (6.00)	0(0)	**<0.001**
NICU admission	32 (32.00)	14(10.70)	**<0.001**
Fetal Distress	8 (8.00)	5 (3.70)	0.131
PROM	9 (9.00)	6 (4.50)	0.130
Preterm birth	22 (22.00)	15 (11.20)	**0.020**
Caesarean section	69(69.00)	74 (55.20)	**0.022**
APGAR 1 min	2 (2.00)	3 (2.20)	0.470
APGAR 5 min	2 (2.00)	0 (0)	**0.004**
Respiratory distress	14 (14.00)	8 (6.00)	0.032
RDS	3 (3.00)	4 (3.00)	0.641
Neonatal death	1 (0)	0(0)	0.427
Hyper bilirubinemia at 72 h first after birth	4 (4.00)	3 (2.20)	0.342

IUFD, intrauterine fetal demise; PIH, Pregnancy induced hypertension; IUGR, Intrauterine growth restriction; PPH, Postpartum hemorrhage; ICU, Intensive Care Unit; NICU, Neonatal intensive care unit; PROM, Premature rupture of membranes; APGAR, appearance, pulse, grimace, activity, and respiration; RDS, Respiratory distress syndrome.

Continuous variables compared with independent *t*-test, categorical variables compared with chi-square test or Fisher’s exact test.

**Table 5:** Univariate and multivariate modified Poisson regression analyses of maternal COVID-19 infected and pregnancy and neonatal outcomes.

*Pregnancy and neonatal outcomes*	*Univariate Crude RR*^*a*^ *(95% CI*^*b*^)	*P value*	*Multivariate Adjusted RR (95% CI)*	*P value*
Gestational diabetes	*1.42(0.75-2.68)*	*0.275*	*1.46 (0.68-3.12)*	*0.324*
Pre-eclampsia	*1.17 (0.43-3.14)*	*0.752*	*1.17 (0.30-4.54)*	*0.811*
IUGR	*0.95(0.31-2.94)*	*0.939*	*0.35 (0.06-1.74)*	*0.199*
Preterm birth	*1.09 (1.01-1.19)*	*0.027*	*0.97 (0.87-1.07)*	*0.602*
Caesarean section	*1.88 (1.08-1.17)*	*0.030*	*1.12 (1.09-1.25)*	*0.033*
Fetal distress	*2.14 (0.72-6.38)*	*0.171*	*4.37 (0.84-22.72)*	*0.079*
PROM	*2.01 (0.73-5.48)*	*0.173*	*1.02 (0.20-5.25)*	*0.974*
NICU admitted	*3.05 (1.72-5.42)*	*0.001*	*2.46 (1.06-5.70)*	*0.034*
Asphyxia at birth	*2.34(1.01-5.39)*	*0.045*	*1.36 (0.42-4.44)*	*0.601*

^a^Risk ratio estimated directly from modified Poisson regression or Firth penalized likelihood method considering rarity of outcome.

^b^CI, confidence interval. The final multivariable models were adjusted for the following risk factors: maternal age, body mass index, gestational age, parity, previous pregnancy problems and comorbidity.

*Significant at *P* value *<*0.05.

In general, for patients diagnosed in our hospital, oxygen therapy, lopinavir/ritonavir, and chloroquine therapy were administered. In total, in our study, 143 (61.11%) of them gave birth by CS. In the unadjusted results, the frequency of CS in women infected with Covid-19 was higher compared to the control group [RR = 1.88 (95% CI 1.08–1.17) P = 0.030]. Similarly, after adjusting for potential confounding factors, a significant difference was observed in terms of type of delivery between the two groups of infected and uninfected women [adjusted risk ratio aRR = 1.12 (95% CI 1.09–1.25) P = 0.030] (Table 5).

Pregnant women with covid-19 had a higher incidence of preterm delivery compared to pregnant women without infection (RR = 1.09 CI 95% (1.01–1.19), P = 0.027), but after adjusting for potential confounders, there was not found a significant difference in the incidence of preterm delivery [aRR = 0.97 CI 95% (0.87–1.07, P = 602)].

Pregnant women with Covid-19 had an unadjusted risk ratio (RR = 3.05, 95% CI (1.72–5.70), P = 0.001) for neonatal intensive care unit (NICU) admission compared to uninfected pregnant women. After adjusting for potential confounding factors, there was a significant difference (aRR = 2.46 CI 95% (1.06–5.42), P = 0.034).

Pregnant women with covid-19 had an unadjusted risk ratio (RR = 2.34 CI 95% (1.01–5.39), P = 0.045) for the incidence of neonatal respiratory distress compared to pregnant women without infection, but after adjusting for potential confounders including **age at the time of the infant’s birth**, there was no significant difference (aRR = 1.36 CI 95% (0.42–4.44), P = 0.601). In addition, there was no significant association between COVID-19 infection and gestational diabetes (GDM), preeclampsia, intrauterine growth restriction (IUGR), premature rupture of membranes (PROM), fetal distress during labor (Table 5).

In our study, due to the limitations availability of diagnostic kits, blood was collected from the umbilical cord only from neonate born to women infected with SARS-CoV-2 after birth and cord clamping. A total of 76 cord blood samples were analyzed, revealing that 16 (21.05%) neonates born to mothers infected with SARS-CoV-2 had positive IgG antibodies, and 4 (5.26%) had positive IgM antibodies. For 97 live infants born to mothers with COVID-19, SARS-CoV-2 RT-PCR was performed on nasopharyngeal samples within the first 24 hours after birth. Nearly all (97.94%) newborns tested negative for SARS-CoV-2 infection. Only 2 neonates (2.06%), tested positive by RT-PCR, and were transferred to the intensive care unit.

## Discussion

We conducted cohort study to assess the symptoms and associations between COVID-19 in pregnancy and maternal and neonatal outcomes that included Such as the risk of viral infection with the progress of pregnancy, the effect of the virus on pregnancy outcomes, the type of delivery, the possibility of vertical transmission and neonatal outcomes. We displayed that women with COVID-19 diagnosis, compared with those without COVID-19 diagnosis, were at significantly increased risk of adverse pregnancy outcomes.

In our study, the incidence of cesarean sections (CS) was notably higher among infected women. However, this finding should be interpreted with caution, as significant confounding factors such as chronic hypertension and diabetes and other comorbidity were also found to be more prevalent in the infected group although were adjusted for in the analysis, potentially influencing their results. Additionally, there was no significant difference between the groups regarding the reasons for CS. our study identified a history of previous CS as the most common reason for cesarean delivery in two groups. In contrast, a review study indicated that fetal distress was the most frequently reported reason for CS among infected women [30]. In many studies, cesarean delivery was more common among women infected with COVID-19 [29,31–33]. But not all studies agree. Steffen et al. (2021) concluded that there were no significant associations between COVID-19 infection during pregnancy and adverse maternal or neonatal outcomes [34]. This difference is probably due to several factors. In their study, only 61 patients were infected with SARS-CoV-2 during their pregnancy, and only two of these patients experienced a severe illness that required hospitalization in the intensive care unit. Only 27.9% of patients experienced moderate to severe symptoms of COVID-19 [29].

We conducted a cohort study to assess the symptoms and associations between COVID-19 in pregnancy and maternal and neonatal outcomes that included things such as the risk of viral infection with the progress of pregnancy, the effect of the virus on pregnancy outcomes, the mode of delivery, the possibility of vertical transmission and neonatal outcomes. We displayed that women with a COVID-19 diagnosis, compared with those without a COVID-19 diagnosis, were at significantly increased risk of adverse pregnancy outcomes.

Our findings revealed that pregnant women infected with COVID-19 had a greater rate of preterm birth significantly. But after adjusting the possible confounding factors including maternal age, body mass index, gestational age, parity, previous pregnancy problems and comorbidity, the association wasn’t significant. In most studies, there is a significant association between COVID-19 infection during pregnancy and preterm birth [15,30,35–37]. In some other studies, there was no significant relationship between premature birth and mother’s infection with covid-19 [29,34]. But in recent study, if sample size was larger, this association would remain significant probably after adjusted potential confounder factors.

In our study, there was a significant association between newborn respiratory distress and infected COVID-19 in pregnancy. The risk of COVID-19 infection in newborn respiratory distress was 2.34 times higher than that of non-COVID-19 pregnant women. After adjusting potential confounding factors from the analysis, the effect size became insignificant [RR = 1.36 (0.42–4.44)]. Recently, El-Atawi et al. (2024), in a systematic review and meta-analysis study, mentioned the risk of newborn respiratory distress in women infected with COVID-19 as 1.96 times compared to uninfected women [37]. Also, Norman et al. (2021) showed COVID-19 infection during pregnancy was associated with a significantly higher risk of neonatal respiratory distress (odds ratio = 1.42) [38].

In our study, we showed that neonates of mothers with infected COVID-19 were associated with a significantly higher risk of being admitted to NICU compared to those of mothers with uninfected COVID-19 until after adjusting for the following risk factors: maternal age, body mass index, gestational age, parity, previous pregnancy problems and comorbidity. Numerous studies have been published on newborns of COVID-19- infected pregnant women [38–41]. Their findings revealed that neonates of mothers infected with COVID-19 had a greater rate of being admitted to a NICU. However, intrauterine transmission of COVID-19 was not identified when infant outcomes of pregnant women infected with COVID-19 were examined [15,30,35].

In a recent study, nearly all newborns tested negative for SARS-CoV-2 infection after birth (97.94%). In agreement with our study, Khoury et al (2020) also reported that 97.5% of newborns tested negative for SARS-CoV-2 infection immediately after birth [33]. Our results align with current evidence that proposes that in spite of the fact that placental and neonatal SARS-CoV-2 transmission may happen [42–45], this vertical transfer from mother to fetus is not common [15,46,47].

A total of 76 cord blood samples, 16 (21.05%) and 4(5.26%) cases of infants born to mothers infected with COVID-19 had positive IgG and IgM antibodies, respectively. The sensitivity of IgM for SARS-CoV-2 reached 70.2% and specificity was 96.2%. The sensitivity of IgG for SARS-CoV-2 reached 96.1% and specificity was 92.4% [48]. We found productive exchange of IgG antibodies from women who were SARS-CoV-2 infection (transfer ratios ≥1.1 in 16 of 76 infants who had seropositive). Our findings are aligned with Flannery et al (2021). They found efficient transfer of IgG antibodies from women who were SARS-CoV-2 seropositive (transfer ratios ≥1.0 in 40 of 72 infants who were seropositive) [44]. Higher maternal antibody concentrations and a better exchange proportion were related with expanding length between onset of maternal infection and time of delivery [49]. In our study, the women had recently experienced a symptomatic infection of COVID-19, so the percentage of antibody transfer from mother to fetus was lower than in the study of Flannery et al. Probably it’s because our pregnant women infected with corona virus-19 were all hospitalized due to the severity of the disease.

### Strengths and limitations

One of the strengths of our study is that the study was planned as a prospective cohort study. Another strength of our study is that we did not classify women who only had positive RT-PCR tests for COVID-19 as infected. Due to the possibility of false negative results in RT-PCR virus, we have considered people with coronavirus infection who also have clinical signs and symptoms of coronavirus infection. The main strength of our study was the careful selection of the exclusion criteria for both groups, especially the control group. That is, in the control group there were those who had negative RT-PCR for COVID-19 and did not have any clinical signs and symptoms of coronavirus infection.

The study’s limitations encompass the collection of samples from a single site, some sample size. Despite having a higher sample size than the majority of previous COVID-19 studies, the small sample size results in a wide confidence interval for rare pregnancy outcomes.

The study was limited because the antibody diagnostic kits for detecting coronavirus in infants at birth were limited, so cord blood was only collected from infants born to women with Covid-19. It would have been preferable to collect umbilical cord blood from all newborns and test it for the presence of antibodies.

## Conclusion

The study’s findings indicate that neonates born to mothers infected with COVID-19 had higher rates of cesarean deliveries and NICU admissions, suggesting that maternal infection may influence delivery methods and immediate health needs of newborns due to complications associated with the virus during pregnancy. However, the study did not find substantial differences in other pregnancy-related complications, such as prematurity or low birth weight, between COVID-19 infected mothers and non-infected mothers. This indicates that while COVID-19 may pose specific risks for surgical delivery and postnatal care, its overall impact on other complications may be less pronounced than previously thought. These results highlight the complex interplay between maternal infection and pregnancy outcomes, indicating that COVID-19 introduces unique risks that may necessitate increased healthcare interventions.

## Supporting information

S1 Checklist STROBE checklist cohort. (DOCX)

S2 File Supporting table. (DOCX)
