# Supplementary material for: The relationship between maternal COVID-19 with fetal and neonatal complications and intrauterine vertical transmission: A cohort study on pregnant women
Source: PLoS One. 2025 Jul 16;20(7):e0326450. doi: 10.1371/journal.pone.0326450 (PMC12266458; doi:10.1371/journal.pone.0326450)
Supplement: S2 File — (DOCX) [file pone.0326450.s002.docx]

**Supplementary Table S1**

### Medications Administered in COVID-19 infected pregnant women in Rohani hospital,Babol, Iran

| **Medication Category** | **Specific Drugs/Therapies** | **Number of Patients** | **Percentage** |
| --- | --- | --- | --- |
| **Oxygen Therapy** | Oxygen supplementation | 24/97 | 24.74% |
| **Antiviral Therapy** | Lopinavir/Ritonavir | 72/97 | 74.22% |
| **Antimalarial Therapy** | Chloroquine | 87/97 | 89.69% |
| **Corticosteroids** | Betamethasone,  Dexamethasone | 9/97 | 9.27% |

Data presented here are representative averages based on available September 2020 to September 2021 at Rouhani Hospital, Iran.
